# Case report: histopathology and molecular pathology analysis on enteric tissue of a COVID-19 patient

**DOI:** 10.1186/s13000-021-01082-7

**Published:** 2021-05-05

**Authors:** Yanling Feng, Dong Zeng, Lvyin Hu, Yuexiang Yang, Shu Song, Yuhan Shi, Jingjing Xu, Wenjuan Guo, Yun Ling, Tangkai Qi, Qingguo Wu, Feng Li, Jilin Cheng, Hongzhou Lu

**Affiliations:** 1 Department of Pathology, Shanghai Public Health Clinical center, Fudan University, Shanghai, China; 2 Department of Clinical Laboratory, Shanghai Public Health Clinical center, Fudan University, Shanghai, China; 3 Department of Infectious Disease, Shanghai Public Health Clinical center, Fudan University, Shanghai, China; 4 Department of Respiratory Medicine, Shanghai Public Health Clinical center, Fudan University, Shanghai, China; 5 Department of Digestive Diseases, Shanghai Public Health Clinical center, Fudan University, Shanghai, China

**Keywords:** COVID-19, SARS-CoV-2, In situ hybridization, Case report

## Abstract

**Aims:**

Patients with COVID-19 can also have enteric symptoms. Here we analyzed the histopathology of intestinal detachment tissue from a patient with COVID-19.

**Methods:**

The enteric tissue was examined by hematoxylin & eosin stain, PAS (Periodic acid–Schiff) staining, Gram staining, Ziehl–Neelsen stain and Grocott’s Methenamine Silver (GMS) Stain. The distribution of CD3, CD4, CK20 and CD68, cytomegalovirus (CMV) and Herpes Simplex Virus (HSV) antigen were determined by immunohistochemistry. In situ hybridization (ISH) of SARS-CoV-2 and Epstein-Barr virus-encoded small RNA (EBER) were also performed.

**Results:**

We observed mucosal epithelium shedding, intestinal mucosal erosion, focal inflammatory necrosis with hemorrhage, massive neutrophil infiltration, macrophage proliferation accompanied by minor lymphocyte infiltration. Fungal spores and gram positive cocci but not mycobacteria tuberculosis were identified. Immunohistochemistry staining showed abundant CD68^+^ macrophages but few lymphocytes infiltration. HSV, CMV and EBV were negative. ISH of SARS-CoV-2 RNA showed positive signal which mostly overlapped with CD68 positivity.

**Conclusions:**

The in situ detection of SARS-CoV-2 RNA in intestinal macrophages implicates a possible route for gastrointestinal infection. Further study is needed to further characterize the susceptibility of enteric cells to SARS-CoV-2 infection.

## Introduction

The coronavirus disease-19 (COVID-19), caused by infection with severe acute respiratory syndrome coronavirus 2 (SARS-CoV-2) [1, 2] can cause diseases of disparate severity. As the knowledge on the clinical manifestations of COVID-19 accumulated, gastrointestinal symptoms such as diarrhea, nausea and vomiting gradually received attention [3]. The viral RNA was found to sustain longer in fecal samples than in respiratory samples [4]. Furthermore, with the expression of ACE2 (angiotensin converting enzyme 2), enterocytes were reported to support SARS-CoV-2 replication and virus shedding [5]. Nevertheless, there has been a lack of direct evidence on the existence of virus within enteric cells. Here we report histological and molecular pathology findings based on the enteric detachment tissues from a COVID-19 patient.

## Material and methods

### Histopathology

Intestinal tissues were fixed with 4% neutral formaldehyde for 6–10 h, routinely dehydrated and embedded with paraffin, 4 μM sections were serially cut on APES (3-Aminopropyltriethoxysilane) coated slides and stained with hematoxylin and eosin (H&E) and observed on a light microscope. PAS (Periodic acid–Schiff) staining, Gram staining, Ziehl–Neelsen stain and Grocott’s Methenamine Silver (GMS) Staining were performed using commercial kits from Baso (Zhuhai, China) according to the manufacturer’s instructions.

### In situ hybridization

The ViewRNA ISH Tissue Assay kit (Affymetrix, CA. USA) was used to detect the plus strand of SARS-CoV-2 RNA with probe set targeting positive strand (target region: nt705–1676, nt4356–5607, nt12808–13,943, nt20571–21,759, nt26645–27,763, nt28212–29,152, Reference LC521925.1) of the viral genome (catalog No. VPNKRHH, Thermo Fisher). The hybridization and amplification procedures were performed according to the protocols provided by the manufacturer with minor modifications. After pretreatment, hybridization and amplification, sections were finally stained with NBT and BCIP (Roche) in developing solution at 37 °C and counterstained with nuclear fast red (Vector Labs, USA). Slides were dehydrated and mounted with Ultra-Clear (Baso, China). In situ detection of EBER was performed using a diagnostic kit from ZSGB-Bio (Beijing, China).

### Immunohistochemistry

Immunohistochemistry staining was performed with Leica Bond-Max autostainer. Sections were routinely dewaxed and rehydrated. After appropriate heat induced antigen retrieval with BOND Epitope Retrival Solution1 or 2, sections were incubated with the primary monoclonal antibody against the CD68 (clone PGM, ZSBio, China), CD3 and CD4, CMV antigen and HSV antigen (Abcam) respectively. Signal was developed with 3,3′-diaminobezdine (DAB). Sections were counterstained with Mayer’s hematoxylin.

### Detection for SARS-CoV-2 nucleic acids

qRT-PCR of SARS-CoV-2 RNA was performed using a commercial kit (DaAn Gene, Guangzhou, China) which targets two regions (orf1a/b and N) for amplification.

## Results

We present a case of a man (Chinese) aged 75 who was admitted into Shanghai Public Health Clinical Center. On arriving at Shanghai from United States by airflight on March 19th 2020, he was identified to have abnormal body temperature (37.7 °C) and was later tested positive for SARS-CoV-2 RNA. His wife, accompanying him on the flight, was diagnosed with COVID-19 1 day later. The patient had high blood pressure for 21 years and diabetes for 5 years. Computed tomography (CT) scan indicated interstitial changes in the lower lobe of right lung. The key laboratory results during his hospitalizations were summarized in Table 1. Blood test results upon admission showed 6.0 × 10^9^/L leukocyte, 1.6 × 10^9^/L lymphocyte, C reactive protein<5 mg/L (Table 1). qRT-PCR of SARS-CoV-2 RNA from the patient’s throat swab showed positive result. He was treated with inhaled IFN-α and hydroxychloroquine. The patient’s condition deteriorated on the 7th day after admission with body temperature of 39 °C and acute respiratory distress. The gastrointestinal symptoms included abdominal pain, abdominal distension and diarrhea but no nausea or vomiting. He was soon intubated and later treated with ECMO (Extracorporeal Membrane Oxygenation). Linezolid (0.6 g q12h) was administered by nasal feeding and Tazobactam and Piperacillin was administered intravenously. Oval-shaped fungi were identified in sputum samples 15 days after admission. On day 27, anal drainage revealed yellow paste-like feces. On day 30, the patient had loose stool of 1700 ml, accompanied by blood mucus. White spots were later found in his oral cavity which was treated with flucanazol capsule. Fecal occult blood was identified on day 33 post admission. Enteroscopic examination was performed to evaluate the degree of intestinal bleeding, which reported intestinal and colonic erosion, ulcer, hyperplasia, bleeding and mucus exudation. Hemostatic drug flushing was performed during enteroscopy. On day 45, the blood test showed 15.07 × 10^9^/L leukocyte, highly abundant neutrophil (97.10% in leukocyte), low lymphocyte counts (0.44 × 10^9^/L), low CD3 T cell count (240 /μl), C reactive protein 117.39 mg/L and prothrombin time 27.50 s. Extremely high level of inflammatory cytokines such as IL-61128.07 pg/ml and IL-8 34.03 pg/ml were reported (Table 1). The patient’s throat swab, feces and bronchial lavage fluid were all tested positive for SARS-CoV-2. CT scans showed bilateral scattered patchy high-density regions and pleural effusion. After intensive care and treatment, the patient finally recovered 60 days after hospitalization and viral RNA was negative.

**Table 1:** Basic clinical parameters during hospitalization

Clinical Variables	Day 2	Day 11	Day 27	Day 42	Day 45	Day 54	Day 71
Leukocyte (10^9^/L)	6.0	15.64	6.62	12.66	15.07	12.25	9.33
Lymphocyte (10^9^/L)	1.6	0.63	0.36	0.76	0.44	0.85	0.50
CD3 T lymphocyte (/ul)	929	239	280	581	240	719	467
Neutrophil (10^9^/L)	4.5	14.58	5.15	9.76	14.63	10.41	8.51
SARS-CoV-2 RNA (−/+)	+	+	+	+	+	+	–
C reactive protein (mg/L)	< 5	111.32	127.75	50.21	117.39	29.41	28.36
IL-6 (pg/ml)	0	387.82	11.26	19.64	1128.07	46.96	15.91
IL-8 (pg/ml)	0.07	52.54	22.95	21.34	34.03	22.88	30.25

**Fig. 1:**
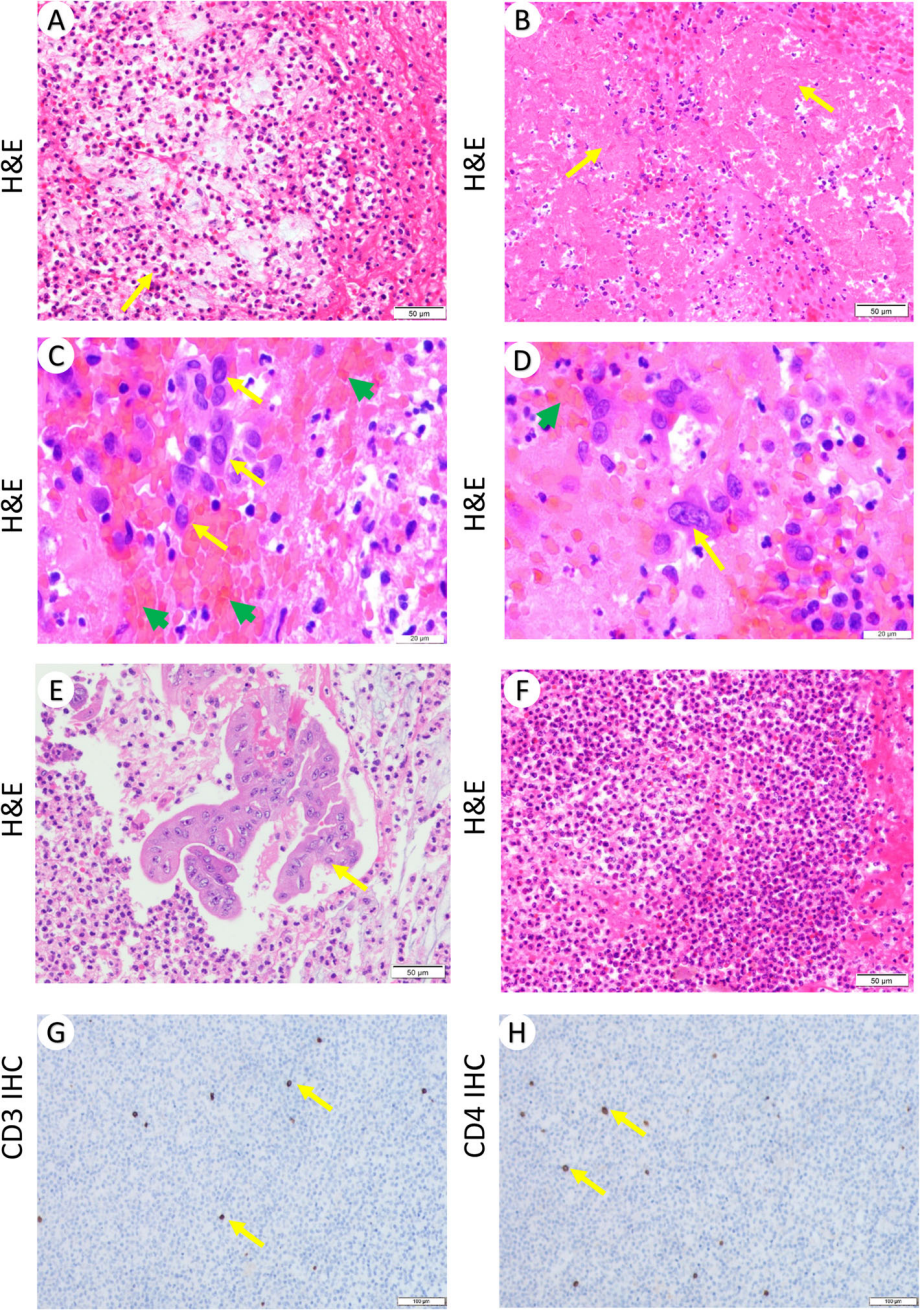
Histopathological findings in the intestinal tissues. **a** Massive neutrophil infiltration accompanied by fibrin, erythrocyte and mucus exudation (200X). **b** focal inflammatory necrosis (200X); **c**-**d** enlarged nuclei and deep staining of macrophages (yellow arrow) with prominent nucleoli, focal hemorrhage accompanied by neutrophil and lymphocyte infiltration (400X). **e** residual glandular epithelial cells with hemorrhage necrosis, fibrin and mucus exudation (200X). **f** Focal purulent inflammation (200X). **g** Immunohistochemistry of CD3 (100X), **h** Immunohistochemistry of CD4 (100X)

**Fig. 2:**
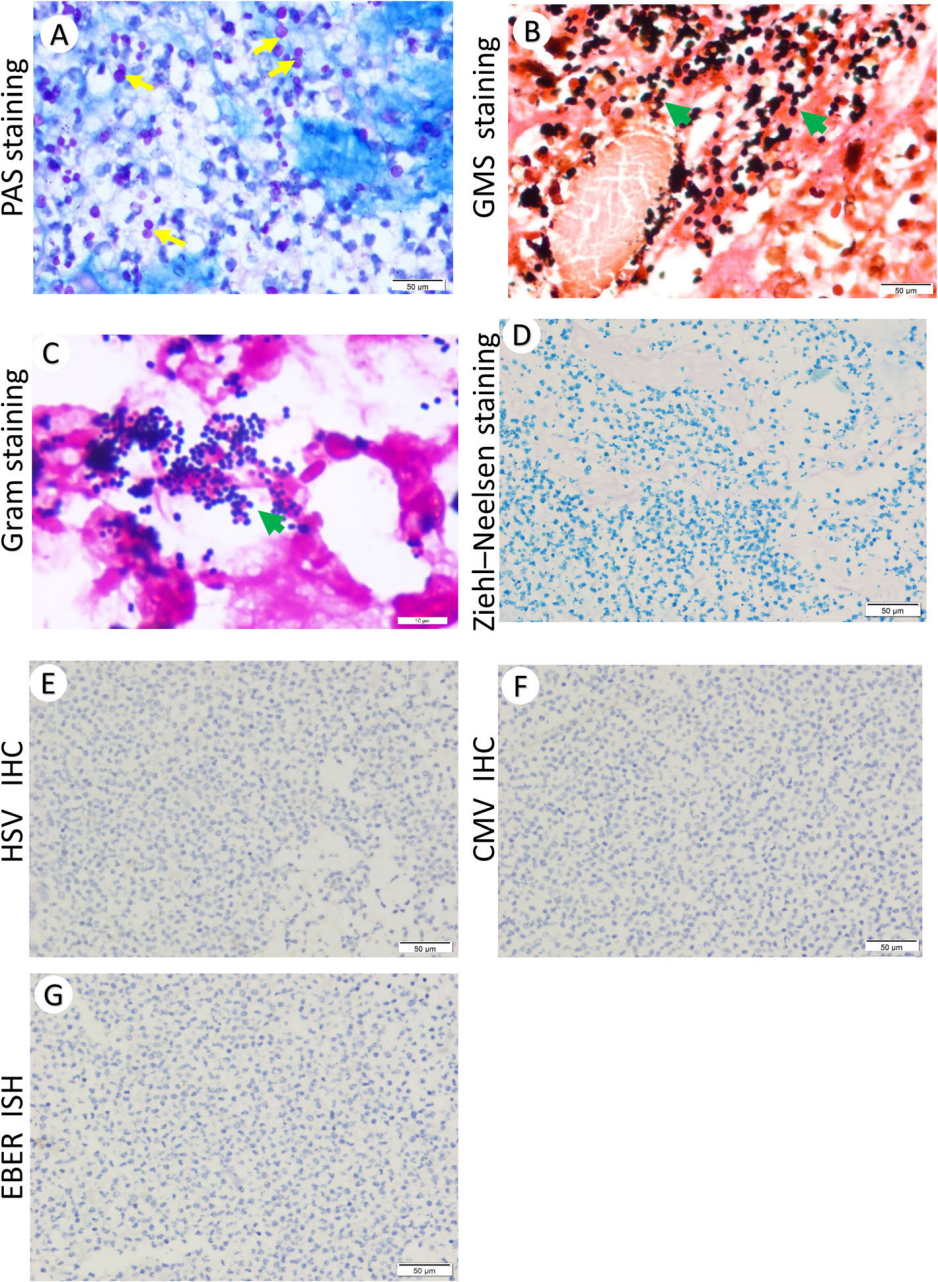
Possible microbiological infections in the intestinal tissue. **a** Fungal spores (purple red, yellow arrows) but without pseudohypha were identified by PAS staining (400X), **b** Fungal spores (black, green arrows) were identified by GMS staining (400X), **c** Gram staining revealed positive cocci (purple, green arrow, 1000X). **d** Ziehl–Neelsen stain (400X) did not identified *Mycobacterium tuberculosis*. Immunohistochemistry of HSV (**e** 400X) and CMV (**f** 400X) and in situ hybridization of EBV (**g** 400X) did not showed signal

The mucous and necrotic tissues in feces were collected on day 47 and 49 after admission for pathology. Three blocks of pale and beige enteric tissues with sizes of 3 × 2 × 1 cm, 4 × 2 × 1.5 cm, 2.5 × 1.5 × 0.8 cm were examined. Under the microscope, we observed massive neutrophil infiltration (Fig. 1a, arrow), focal inflammatory necrosis (Fig. 1b, arrow) and hemorrhage (Fig. 1c-d, green arrow), mucosal epithelium shedding and erosion (Fig. 1e, arrow), accompanied by abscess (Fig. 1f). Macrophage infiltration (Fig. 1c-d, yellow arrow) and few lymphocytes (Fig. 1g-h, arrow) were found. To examine the possible microbiological infection, PAS staining and GMS Staining as well as Gram staining were performed. Significant number of round and oval-shape fungal spores (Fig. 2a-b, arrow) but not pseudohypha were observed. Mass spectrometry of isolated colonies identified them to be Candida galbrata. Gram positive cocci (Fig. 2c, arrow) were also found. No mycobacteria were identified using Ziehl–Neelsen stain (Fig. 2d). Immunohistochemistry for HSV (Fig. 2e) and CMV antigens (Fig. 2f) were negative. In situ hybridization of EBER RNA was negative (Fig. 2g).

**Fig. 3:**
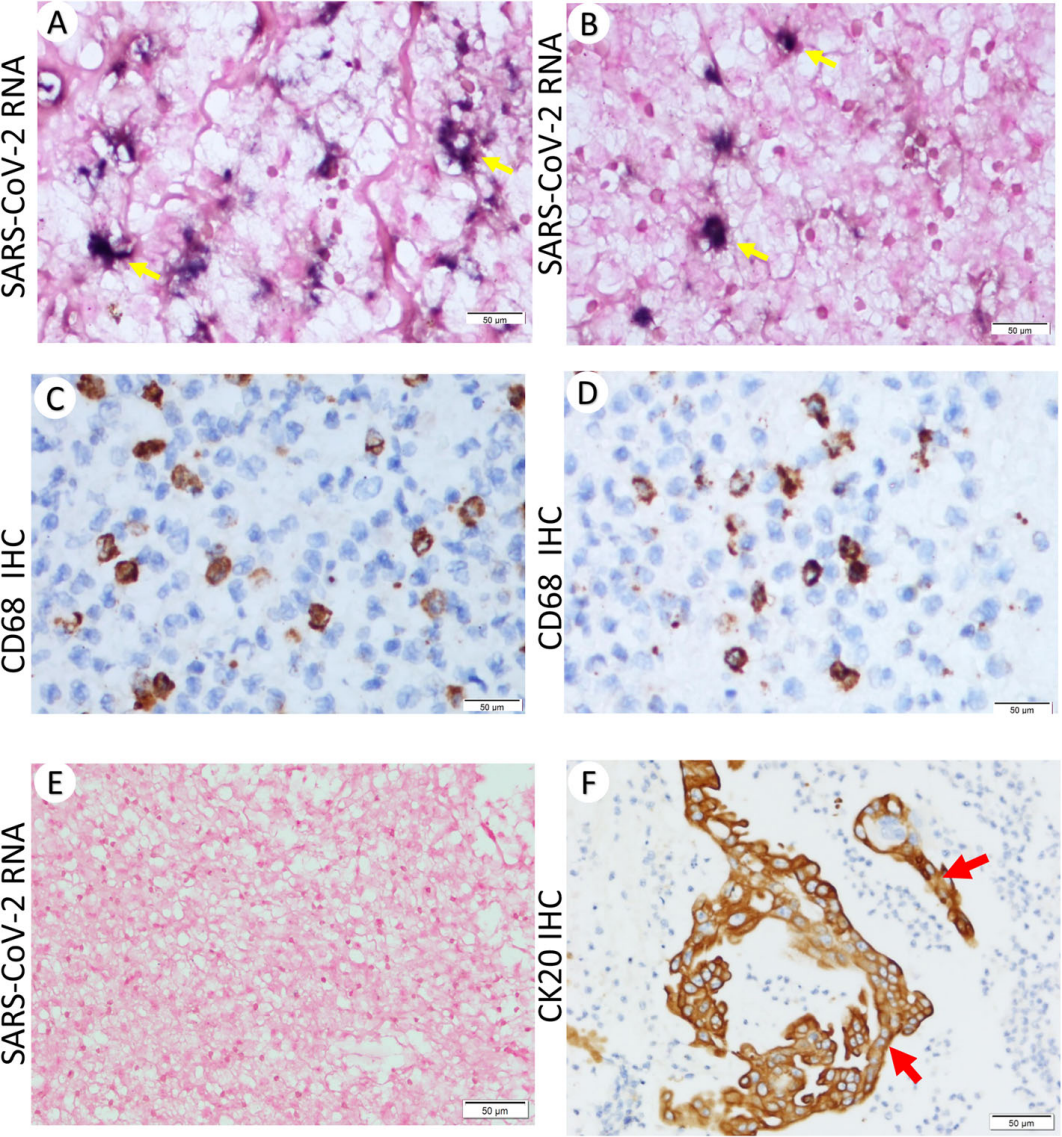
In situ detection of SARS-CoV-2 RNA in intestinal macrophages. **a**, **b** In situ hybridization of SARS-CoV-2 RNA (400X) **c**, **d** Immunohistochemistry of CD68 (400X). **e** in situ hybridization of SARS-CoV-2 RNA in enteric tissue of uninfected individual, **f** Immunohistochemistry of CK20 (400X). **a** and **c**, **b** and **d** are from same regions of adjacent sections

Next, we performed in situ hybridization of SARS-CoV-2 RNA. The blue purple signal was observed in the cytoplasm (Fig. 3a-b, arrow). The specificity of the in situ assay was confirmed by the lack of background in enteric tissues collected from uninfected patients (Fig. 3e). Immunostaining of the adjacent sections of the same tissues showed a large number of CD68^+^ positive macrophages (Fig. 3c-d). On the other hand, CK20 positive (epithelial) cells were very rare (Fig. 3f, arrow) in these tissues. No viral RNA positive cells were found to reside in these cells.

## Discussion

Although SARS-CoV-2 shares around 80% sequence similarity with SARS-CoV, its clinical manifestations are far more variable, ranging from asymptomatic carrier to life-threatening respiratory failure. The risk factors associated with disease severity were host factors such as older age and comorbidities [6]. Apart from pulmonary disease, diarrhea was consistently documented as a minor manifestation of COVID-19. Importantly, the percentage of patients showing GI symptoms increases during hospitalization [7]. In this reported case, the pathological findings indicated excessive inflammatory responses such as neutrophil infiltration, macrophage hyperplasia which resulted in excessive intestinal damages such as mucosal epithelium shedding erosion and focal inflammatory necrosis. Additional staining revealed the colonization of fungi and pathogenic Gram-positive bacteria. Indeed, severe SARS-CoV-2 infection can cause systemic inflammatory response which could significantly disturb the homeostasis of the gut microbiota [8, 9] and facilitate the growth of pathogenic microbes. In the case reported here, GI symptom was observed on day 27 after admission and coincided with fungal and bacterial infection in the intestine. The older age and co-existing conditions may further deteriorate the intestinal micro-environment.

Apart from the impact of altered intestinal microflora, SARS-CoV-2 may play a direct role in the gastrointestinal symptoms. Transcriptomic and proteomic profiling in various human tissues indicated that intestinal tissue expresses the most abundant ACE2 [10]. Indeed, productive infection was reported in primary human enteroid culture [5]. Three potential mechanisms were thought to explain the spread of SARS-CoV-2 into the gut. 1), The viral particles released into circulation could spread to other susceptible organs; 2), SARS-CoV-2 could be consistently detected in posterior oropharyngeal saliva most probably shed by upper respiratory cells [11] which may be responsible for a large part of viral input into the GI tract and hence enterocyte infection; 3), Alveolar macrophage, which also expresses ACE2, could be a carrier of this virus [12] and facilitate extra-pulmonary infection via macrophage trafficking. Indeed, Wang et al. reported the positive staining of viral nucleocapsid protein in not only pneumocytes but also alveolar macrophages [12]. In the case reported here, the viral RNA signal was found to reside in tissues with abundant infiltrated macrophages which might support the third hypothesis. Nevertheless, due to the very few intestinal epithelial cells detected in the tissues, the possibility of enterocyte infection in vivo cannot be conclusively ruled out.

Taken together, we reported comprehensive pathological analysis of one COVID-19 case with severe secondary gastrointestinal disease. These serious manifestations were the combined result of a series of microbiological insults, e.g., bacterial and fungal colonization, as a result of prolonged respiratory failure in addition to enteric viral infection. The results of molecular pathology analysis suggested the role of macrophage in spreading SARS-CoV-2 into the gastrointestinal tract. Additional study is needed to clarify the in vivo viral susceptibility profile of intestinal cell types.

## Data Availability

The relevant data and materials pertaining to this study are available upon request.

## Abbreviations

COVID-19: Coronavirus disease-19
ISH: In situ hybridization
SARS-CoV-2: Severe acute respiratory syndrome coronavirus 2
CMV: Cytomegalovirus
HSV: Herpes Simplex Virus
EBER: Epstein-Barr virus-encoded small RNA
GMS: Grocott’s Methenamine Silver
ACE2: Angiotensin converting enzyme 2
CT: Computed tomography
GI: Gastrointestinal
CK7: Cytokeratin 7
CK20: Cytokeratin 20
